# Frequency of Cerebral Aneurysm in patients with subarachnoid hemorrhage on CT Cerebral Angiography

**DOI:** 10.12669/pjms.40.9.8653

**Published:** 2024-10

**Authors:** Sana, Shaista Shoukat, Sumera Tabassum, Haania Shahbaz

**Affiliations:** 1 Sana, Radiologist, Department of Radiology, Jinnah Postgraduate Medical Centre, Karachi, Pakistan; 2 Shaista Shoukat, Associate Professor, Department of Radiology, Jinnah Postgraduate Medical Centre, Karachi, Pakistan; 3 Sumera Tabassum, Associate Professor, Department of Radiology, Jinnah Postgraduate Medical Centre, Karachi, Pakistan; 4 Haania Shahbaz, Research Fellow, Jinnah Postgraduate Medical Centre, Karachi, Pakistan

**Keywords:** Subarachnoid haemorrhage, CT cerebral angiography, Cerebral aneurysm

## Abstract

**Objective:**

To determine the frequency of cerebral aneurysm in patients with subarachnoid haemorrhage on CT cerebral angiography.

**Methods:**

This prospective cross-sectional study was conducted at Department of Radiology, JPMC, Karachi from 5^th^ June 2022 to 30^th^ January 2023. Total 176 patients with subarachnoid haemorrhage irrespective of gender were selected. CT angiography of cerebral vessels was performed. Cerebral aneurysm was noted as per operational definition and recorded.

**Results:**

Age range was from 18 to 60 years. Mean age was 39.516±6.77 years, Mean BMI 29.630±3.08 Kg/m2. and mean duration of symptoms was 7.721±2.40 days. Male patients were 80.1% and females were 19.9%. Cerebral aneurysm was observed in 91.4% female and 78.7% male patients. Aneurysmal percentage was slightly higher in younger (18-40 years) age group (84.2% vs 80.4%) as compared to older (40-60) age group but statistically not significant. Although total percentage of female was quite less 19.9% (n=35 vs n=141 male), in this lesser female percentage, aneurysmal detection was higher as compared to male gender (91.4% vs 78.7%). Distribution of aneurysms according to vessel involved was; 28.7% along anterior communicating artery, 28.3% related to middle cerebral artery and, 22.9% were along posterior communicating vessel. Rest was distributed among other vessels including tip of basilar artery.

**Conclusion:**

In Our study aneurysms was seen 81.3% in patients of subarachnoid hemorrhage having CT angiography at our center in Karachi Pakistan.

## INTRODUCTION

Subarachnoid haemorrhage (SAH) occurs in most cases due to rupture of Cerebral aneurysms.^1^,^2^ *Subarachnoid hemorrhage* is labeled when a patient presents with sudden severe headache ‘and’ on CT brain scan there is hyperdense material filling the subarachnoid space around the circle of Willis and/or in the Sylvian fissure. While *Cerebral aneurysm* is defined as a well-defined round, slightly hyperattenuating lesion on CT angiography of cerebral vessels.

Cerebral aneurysms develop at weak points along the arteries of brain. These vary in size from less than 0.5 mm to greater than 25 mm.^3^ Shape of Most of the aneurysms is saccular (berry), These have a thin or sometimes absent tunica media. Internal elastic lamina is totally absent or fragmented. In small percentage Fusiform (circumferential) or mycotic (infectious) aneurysms are also reported. of cases.^1^ Most unruptured cerebral aneurysms are seen incidentally on imaging for some other indication and are termed “silent” and even these silent unruptured are seen during autopsy. Toth G et al. in his article mentioned that more than 80% of aneurysms are found in the anterior circulation and among these most are seen at junctions, or where arteries divide along the circle of Willis.^1^

High-risk individuals for SAH may be screened according to the modality available among conventional angiography, CTA i.e., computerized tomographic angiography or magnetic resonance angiography (MRA).^4^ However, detection of aneurysm becomes much more important, when some patient presents with SAH. As aneurysm being a common cause and fear of rebleed of aneuysm being well recognized phenomenon. It necessitates its localization for surgical treatment. While confirming the diagnosis of SAH in suspected case of SAH, a plane CT head without contrast, with or without a lumbar puncture (LP), is quite reliable practice^5^ for presence of SAH. CTA as compared to conventional angiography is a quite non-invasive method, and is gaining acceptance widely and recommended for detection\presence and location of intracranial aneurysms.^6^ Lot of improvement has been done recently in technology being used in multi-detector CTA. As a result of these improvements the sensitivity and specificity of CTA for finding and localizing of intracranial aneurysms is said to be comparable to that of DSA (digital subtraction angiography).^6^

While comparing with conventional angiography regarding ease of performance, it is said that CTA is done without dedicated angiographic team as needed in conventional angiography. CTA is faster, and easy to perform and obtain results. Stroke and other risks are also at lower level. Furthermore, it is clearly less invasive

There is paucity of data in this issue from our country. Results of international studies cannot be generalized on our local population due to possible variability in frequency and location of cerebral aneurysm in patients with subarachnoid haemorrhage in different populations.^7^,^8^ Therefore this study has been planned in our tertiary medical center of metropolitan city Karachi Pakistan for recording data in our set of population.

Objective of the study was “to determine the frequencies of different locations of cerebral aneurysm in patients with subarachnoid haemorrhage on CT angiography of cerebral vessels and to compare the results with international studies and contribute to the international literature by locally produced data in Karachi Pakistan”.

## METHODS

This prospective cross sectional study was conducted at Department of Radiology, JPMC, Karachi from 5th June 2022 to 30th January 2023. To calculate sample size 95% CI (confidence interval) and margin of error was kept 3%, after putting Expected frequency of cerebral aneurysm of 4.3% in cases of SAH, in sample size calculator on-web (https://www.calculator.net/sample-size-calculator.html) sample size was determined to be 176. A total of 176 patients of both gender of age from 18 to 60 with diagnosis of subarachnoid haemorrhage on CT scan were included in the study. Patients were selected using “non-probability consecutive sampling technique”.

### Ethical approval

The study was “approved by the Ethical Review Board of Jinnah Postgraduate Medical Center” (letter no: F2-81/2021- Genrl /61735/JPMC dated: June 9, 2021)”.

Patients having History of “allergic reaction to contrast dye or iodine allergy”, or history of allergic airway disease and patient having daily medication or patients having serum creatinine level *≥* 1.4 mg/dL by laboratory test were not selected for the study.

### Data collection procedure:

Patients referred from medical wards to Department of Radiology, Jinnah Postgraduate Medical Centre, fulfilling the inclusion criteria Karachi were included in the study. Base line demographic information of selected patients (age, gender, BMI with formula (Kg/m2) and duration of symptoms) were recorded on purpose built proforma by a resident doctor. Informed consent was taken from patients or relatives if patient unconscious, ensuring confidentiality. They were informed that except rare risk of allergic reaction to contrast needed for detection of aneurysm and further management, no additional risk is involved for including in this study.

Initially patients had a “non-contrast Head CT using a multidetector CT scanner, with 3-mm cuts through the posterior fossa and 5-mm cuts through the rest of the brain”. On finding evidence of SAH in plain CT scan brain, CT angiography of cerebral vessels was performed. 100 mL of iodixanol 320 mg/mL preparation was injected at a rate of 4 mL/s. For injecting an 18-gauge catheter already passed and secured was used. All CTAs were scanned in 2.5-mm cuts from the inner table of the skull vertex to the C1/C2 level.

Cerebral aneurysm presence was noted and its features and location were recorded. For vascular anatomy, three-dimensional rotational reconstructions of vessels were prepared from these cuts according to the need of study by two radiologists having more than three years post fellowship experience. Data was recorded in purpose built proforma

### Data analysis:

Software program SPSS 23 was used for data analysis. The qualitative variables like gender and cerebral aneurysm presence/absence were processed by calculating frequency and percentages. The quantitative variables like age, BMI and duration of symptoms were processed by calculating mean and standard deviation/Med (IQR). Cerebral aneurysm was stratified for age, gender, duration of symptoms and BMI. Post stratification chi square test/Fisher’s Exact test was applied, p ≤0.05, as standard practice, was marked as statistically significant.

## RESULTS

Age of participants varied from 18 - 60 years with mean age 39.516±6.77 years. Mean BMI 29.630±3.08 Kg/m2 and mean duration of symptoms 7.721±2.40 days. Male patients were 80.1% and females were 19.9%. Cerebral aneurysm was observed in 143 patients out of 176 (81.2%) patients, while not seen in 33(18.8%) cases.

Although total percentage of female was quite less 19.9% (n=35 vs n=141 male), in this lesser female percentage, aneurysmal detection was quite higher as compared to male gender (91.4% vs 78.7%) Table-I.

**Table-I:** Stratification of Cerebral Aneurysm with respect to gender.

Gender	Cerebral Aneurysm		p-value

	Yes	No	
Male	111(78.7%)	30(21.3%)	0.085
Female	32(91.4%)	3(8.6%)	
	143(81.2%)	33(18.8%)	

Similarly aneurysmal percentage was slightly higher in younger (18-40 years) age group (84.2% vs 80.4%) as compared to older (40-60) age group but statistically not significant, but frequency of SAH was quite higher in older (40-60) age group as compared to younger (18-40 years) age group (Table-II). Distribution of aneurysms according to vessel involved was as follow. 28.7% along anterior communicating artery, 28.3% related to middle cerebral artery and, 22.9% were along posterior communicating vessel. Rest was distributed among other vessels including tip of basilar artery.

**Table-2:** Frequency of SAH in age groups.

Age group	n	Percentage
18-40	38	21.59 %
40-60	138	78.41 %

Total	176	100 %

## DISCUSSION

Among 176 patients in this study, 38 (21.6%) were aged 18 to 40 years and 138 (78.4%) were 41 to 60 years; 141 (80.1%) were male and 35 (19.9%) were female; 126 (71.6%) were obese and 50 (28.4%) patients were non-obese. Moreover, only in 143 (81.3%) patients’ aneurysms was seen while in 33 (18.8%) individuals no cerebral aneurysms were found.

In study by Roquer J et al. out of 476 consecutive patients with spontaneous subarachnoid hemorrhage, 347 patients (72.9%) were found to be having aneurysm as a cause of SAH.^9^ In another study in Hazara Division Pakistan by Alam S et al.^10^ aneurysm was found in 75.63% cases of SAH which is comparable to our study, but male to female incidence was found reversed (31.93% male vs 43.70% female of total of SAH cases).

In study by Schertz M et al.^11^ similar results were seen with some differences. Their study population was 121 individuals with diagnosis of SAH, among these in 96, the cause determined was aneurism (79.3%) comparable to 81.3 % with aneurysm in our study. However, in Schertz’s study gender wise prevalence was reversed in favor of female preponderance (71.1% female versus 28.9%male, p<0.001), while in our study male predominance was recorded (80.1% male vs 19.9% female). Average age recorded by Schertz was 57.1 years compared to 39.5 years in our study. Crude incidence recorded by him was “4.36 per lac population” with (CI 95%). Ronne-Engström E et al., studied on 615 patients with SAH, from 2007 to 2011.^12^ Among these 615, 448 patients (72.8%) were found having aneurysm.

In study by Song JP et al. out of 2562 individuals with SAH, 81.4% were concluded having aneurysm and 18.6% having no aneurysm.^13^ Frequency of complications in patients gone through DSA (digital subtraction angiography) was 3.9%. Among patients with aneurysms 321 cases (15.4%) were having multiple aneurysms. “Maximum aneurysms were found along anterior communicating artery (30.1%) followed by posterior communicating artery (28.7%), and then middle cerebral artery (15.9%)”.

Among 365 (76.5%) of patients of SAH in which aneurysm was not found by DSA modality, 62 were having PNSAH (peri-mesencephalic non-aneurysmal SAH). Out of these 365, for 252 patients CTA was done or DSA was repeated. In patients with PNSAH (peri-mesencephalic non-aneurysmal SAH), which were 45 in number, repeated examination was negative again but among remaining 207 of 252, in 28 (13.5%) cases aneurysm was found on repeated examination. Among NASAH (non-aneurysmal SAH) commonest cause concluded was arteriovenous malformation (AVM, 7.5%) followed by Moyamoya disease (7.3%), arterial stenosis, (2.7%), and arteriovenous fistula (2.3%).^13^

In another study by Elhadi et al. intracranial aneurysms were found at a higher value (88.9%) in patients with SAH.^14^ Kelliny in his study reported frequency of aneurysm in SAH as 66.3% using CT cerebral angiography modality.^8^ In cases of SAH “where aneurysm rupture is the cause, there is high morbidity and mortality rate .^8^ It is said that mostly these aneurysms are acquired one. Persons with certain risk factor are known to have higher incidence.^1^,^2^ These risk factors are smoking, hypertension, advanced age, atherosclerosis and alcohol abuse. In addition to these endocarditis, cocaine use, trauma and tumors are also implicated.^15^ In patients having family history of aneurysm, genetic factors are also narrated.^15^ Some hereditary diseases include Ehlers-Danlos syndrome (EDS), polycystic kidney (ADPKD) disease, Fibromuscular dysplasia, AVM (arteriovenous malformations), coarctation of aorta and tuberous sclerosis.^14^

Although many unruptured aneurysms are seen incidentally when imaging is done for some other indication, routine screening is mostly not done^4^, however persons with strong family history may be recommended for screening with conventional angiography, CTA (computerized tomographic angiography) or MRA (magnetic resonance Angiography).

CTA (computerized tomographic angiography) being quite non-invasive modality, is being established as modality of choice in the screening of persons with strong family history of having intracranial aneurysms.^6^ Effects of Blood groups in cases of aneurysmal bleed and delayed cerebral ischemia has also been studied but still needs more elaboration.^16^ For cases of SAH, relevant artery having aneurysmal bleed is managed by clipping of relevant artery or by endovascular coiling or embolization.^17^-^19^ Comparison and pros and cons of these methods are being reviewed as multiple studies are going on in various centers.^20^ Studies are also going on to prevent vasospasm in cases of SAH, and new agents are being tried in combination with nimodipine to help manage cases of SAH.^21^

### Limitations:

CTA does not detect all aneurysm especially smaller ones having diameter less than 4mm as studies employing DSA (Digital subtraction angiography) have revealed. CTA also showed lower sensitivity for posterior circulation aneurysms in comparison with DSA. For these reasons DSA is recommended for patients having CTA negative SAH.^22^

## CONCLUSION

The prevalence of intracranial aneurysms was 81.3% in individuals presenting with spontaneous subarachnoid haemorrhage in tertiary care center of metropolitan city of Karachi, Pakistan. More studies are suggested to follow the cases of aneurysmal bleed having gone through different management modalities such as clipping, coiling or embolization.

### Authors’ Contributions:

**S:** Conceived and designed the study, included the patients. Contributed to drafting and revising of article, critical appraisal of findings with literature, final approval. Responsibility for integrity of work.

**SS and ST:** Final interpretation of CT scan findings, contributed to drafting and revising of article, contributed to critical appraisal of findings with literature, contributed to final approval.

**ST:** Statistical analysis, Review

**HS:** Contributed to Patient enrollment, proforma filling, data collection, drafting and SPSS data entry and processing, statistical analysis and final approval and helped in revising the article and drafting.

All authors accept the responsibility for the integrity of the research.
